# The genome sequence of the bootlace worm, *Lineus longissimus *(Gunnerus, 1770)

**DOI:** 10.12688/wellcomeopenres.17193.1

**Published:** 2021-10-14

**Authors:** Dominic Kwiatkowski, Mark Blaxter, Darwin Tree of Life Barcoding collective, Wellcome Sanger Institute Tree of Life programme, Wellcome Sanger Institute Scientific Operations: DNA Pipelines collective, Tree of Life Core Informatics collective, Darwin Tree of Life Consortium

**Affiliations:** 1 Wellcome Sanger Institute, Cambridge, CB10 1SA, UK

**Keywords:** Lineus longissimus, bootlace worm, genome sequence, chromosomal

## Abstract

We present a genome assembly from an individual *Lineus longissimus *(the bootlace worm; Nemertea; Pilidiophora; Heteronemertea; Lineidae). The genome sequence is 391 megabases in span. The majority of the assembly is scaffolded into 19 chromosomal pseudomolecules.

## Species taxonomy

Eukaryota; Metazoa; Spiralia; Lophotrochozoa; Nemertea; Pilidiophora; Heteronemertea; Lineidae; Lineus; *Lineus longissimus* (Gunnerus, 1770) (NCBI:txid88925).

## Introduction

*Lineus longissimus* (Lineidae, Heteronemertea, Nemertea) is a predatory ribbon worm, renowned as being the longest animal in the British and Irish biota, as individual specimens can exceed 25 m when fully extended. Their extensibility results in part from their unsegmented body form, where the coleom is limited to a rhynchocoel associated with the eversible proboscis. Phylum Nemertea is placed as sister to either Mollusca or Annelida within Eutrochozoa in the Spiralia ( Struck & Fisse, 2008). Nemertea includes only 1200 described species worldwide that play important ecological roles in littoral and benthic communities ( Gibson, 1972). Nemerteans have been studied for their ability to regenerate body parts ( Zattara *et al*., 2019) and the potent venom neurotoxins secreted from the glandular epithelium of the proboscis ( Stricker & Cloney, 1983). The nemertide alpha-1 toxin from *L. longissimus* shows promise as an insecticide ( Bell *et al*., 2021).

## Genome sequence report

**Figure 1.**
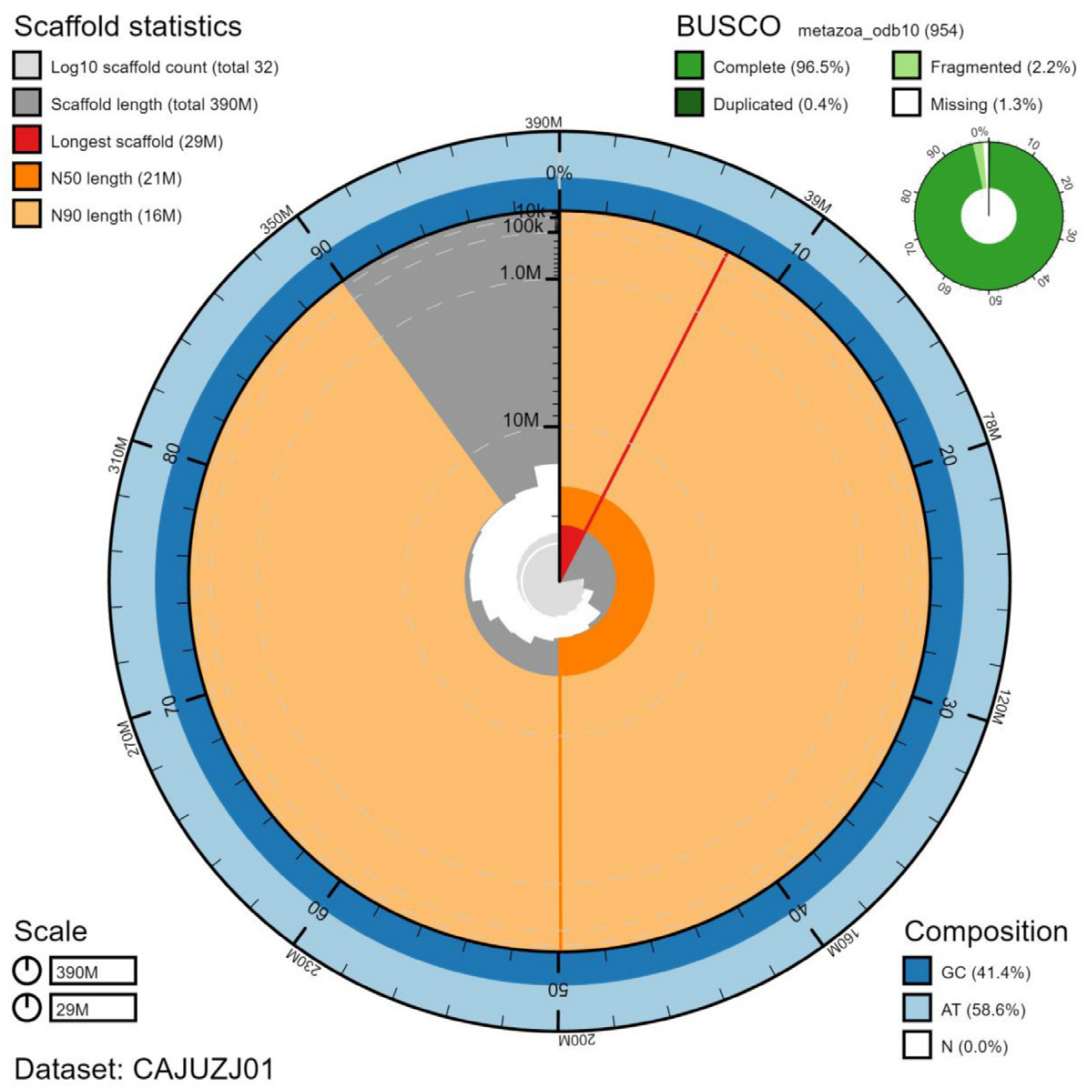
Genome assembly of *Lineus longissimus*, tnLinLong1.1: metrics. The BlobToolKit Snailplot shows N50 metrics and BUSCO gene completeness. An interactive version of this figure is available at https://blobtoolkit.genomehubs.org/view/tnLinLong1.1/dataset/CAJUZJ01/snail.

The genome was sequenced from a single *L. longissimus* of unknown sex collected from White Bay, Great Cumbrae, North Ayreshire, Scotland (latitude 55.790409, longitude -4.908826). A total of 79-fold coverage in Pacific Biosciences single-molecule long reads and 107-fold coverage in 10X Genomics read clouds were generated. Primary assembly contigs were scaffolded with chromosome conformation Hi-C data. Manual assembly curation corrected 67 missing/misjoins and removed 5 haplotypic duplications, reducing the assembly length by 0.46% and the scaffold number by 63.10%, and increasing the scaffold N50 by 53.76%. The final assembly has a total length of 391 Mb in 32 sequence scaffolds with a scaffold N50 of 21 Mb ( Table 1). Of the assembly sequence, 99.81% was assigned to 19 chromosomal-level scaffolds, representing 19 autosomes (numbered by sequence length) ( Figure 1– Figure 4; Table 2). The assembly has a BUSCO ( Simão *et al*., 2015) v5.1.2 completeness of 96.5% using the metazoa_odb10 reference set. While not fully phased, the assembly deposited is of one haplotype. Contigs corresponding to the second haplotype have also been deposited.

**Table 1.** Genome data for Lineus longissimus, tnLinLong1.1.

*Project accession data*	
Assembly identifier	tnLinLong1.1
Species	*Lineus longissimus*
Specimen	tnLinLong1
NCBI taxonomy ID	NCBI:txid88925
BioProject	PRJEB45185
BioSample ID	SAMEA7522833
Isolate information	Unknown sex, anterior/mid/ posterior body
*Raw data accessions*	
PacificBiosciences SEQUEL II	ERR6412039, ERR6436382, ERR6436383
10X Genomics Illumina	ERR6054915-ERR6054918
Hi-C Illumina	ERR6054914
*Genome assembly*	
Assembly accession	GCA_910592395.1
*Accession of alternate haplotype*	GCA_910592375.1
Span (Mb)	391
Number of contigs	109
Contig N50 length (Mb)	10
Number of scaffolds	32
Scaffold N50 length (Mb)	21
Longest scaffold (Mb)	29
BUSCO * genome score	C:96.5%[S:96.1%,D:0.4%],F:2.2%,M:1.3%,n:954

*BUSCO scores based on the metazoa_odb10 BUSCO set using v5.1.2. C= complete [S= single copy, D=duplicated], F=fragmented, M=missing, n=number of orthologues in comparison. A full set of BUSCO scores is available at https://blobtoolkit.genomehubs.org/view/tnLinLong1.1/dataset/CAJUZJ01/busco.

**Figure 2.**
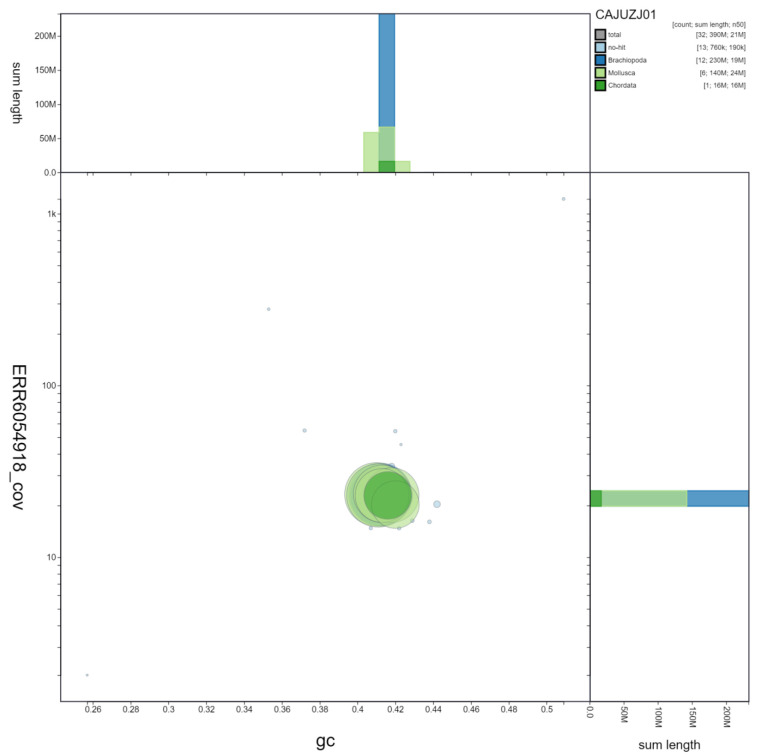
Genome assembly of Lineus longissimus, tnLinLong1.1: GC coverage. BlobToolKit GC-coverage plot. Scaffolds are coloured by phylum. Circles are sized in proportion to scaffold length. Histograms show the distribution of scaffold length sum along each axis. An interactive version of this figure is available at https://blobtoolkit.genomehubs.org/view/tnLinLong1.1/dataset/CAJUZJ01/blob. It should be noted that the tnLinLong1 genome is the first of its phylum (Nemertea) to be assembled, meaning there are few/no other related sequences for BlobToolKit to call in INSDC databases. The resultant identification of Mollusca, Chordata and Brachiopoda sequences here reflects the divergence of Nemertea from other phyla.

**Figure 3.**
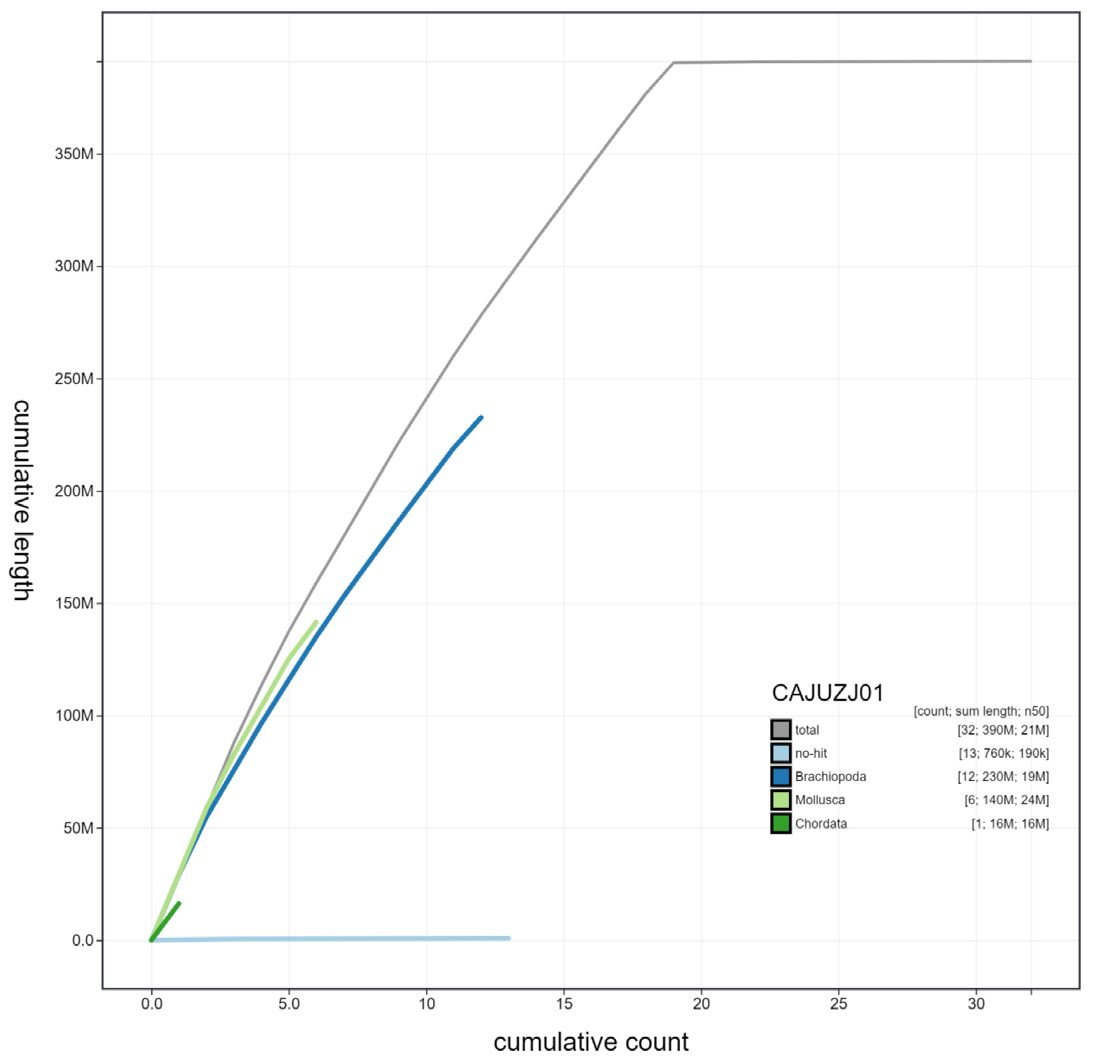
Genome assembly of Lineus longissimus, tnLinLong1.1: cumulative sequence. BlobToolKit cumulative sequence plot. The grey line shows cumulative length for all chromosomes. Coloured lines show cumulative lengths of chromosomes assigned to each phylum using the buscogenes taxrule. Since the tnLinLong1 genome is the first of its phylum (Nemertea) to be assembled, there are few/no other related sequences for BlobToolKit to call in INSDC databases. The resultant identification of Mollusca, Chordata and Brachiopoda sequences here reflects the divergence of Nemertea from other phyla. An interactive version of this figure is available at https://blobtoolkit.genomehubs.org/view/tnLinLong1.1/dataset/CAJUZJ01/cumulative.

**Figure 4.**
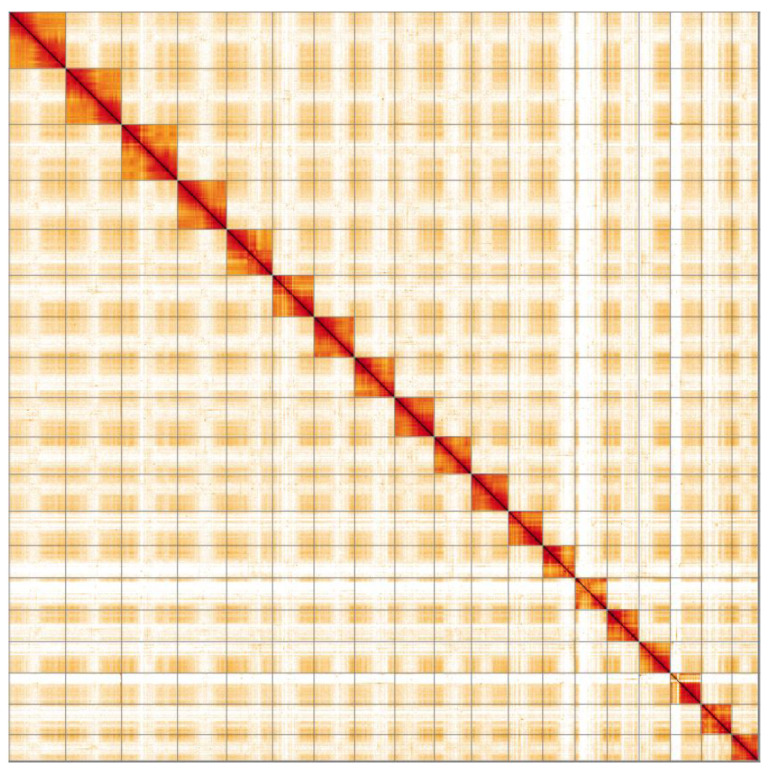
Genome assembly of Lineus longissimus, tnLinLong1.1: Hi-C contact map. Hi-C contact map of the tnLinLong1.1 assembly, visualised in HiGlass.

**Table 2.** Chromosomal pseudomolecules in the genome assembly of *Lineus longissimus*, tnLinLong1.1.

INSDC accession	Chromosome	Size (Mb)	GC%
OU342992.1	1	29.45	41.0
OU342993.1	2	29.19	41.1
OU342994.1	3	29.08	41.2
OU342995.1	4	25.60	41.2
OU342996.1	5	23.94	41.3
OU342997.1	6	21.66	41.8
OU342998.1	7	21.16	41.3
OU342999.1	8	20.96	41.3
OU343000.1	9	20.55	41.4
OU343001.1	10	19.41	41.4
OU343002.1	11	19.19	41.5
OU343003.1	12	17.98	41.6
OU343004.1	13	16.85	41.6
OU343005.1	14	16.76	41.5
OU343006.1	15	16.29	42.0
OU343007.1	16	16.53	41.5
OU343008.1	17	16.30	41.6
OU343009.1	18	15.89	41.4
OU343010.1	19	13.63	41.7
OU343011.1	MT	0.02	35.3
-	Unplaced	0.74	42.5

## Methods

A single *L. longissimus* of unknown sex was collected from White Bay, Great Cumbrae, North Ayreshire, Scotland (latitude 55.790409, longitude -4.908826) by Dominic Kwiatkowski, Wellcome Sanger Institute (WSI) and preserved on dry ice prior to transfer to the WSI.

DNA was extracted from midbody tissue at the Tree of Life laboratory, WSI. The tnLinLong1 sample was weighed and dissected on dry ice with tissue set aside for Hi-C sequencing. Samples were cryogenically disrupted to a fine powder using a Covaris cryoPREP Automated Dry Pulveriser, receiving multiple impacts. Fragment size analysis of 0.01-0.5 ng of DNA was then performed using an Agilent FemtoPulse. High molecular weight (HMW) DNA was extracted using the Qiagen MagAttract HMW DNA extraction kit. Low molecular weight DNA was removed from a 200-ng aliquot of extracted DNA using 0.8X AMpure XP purification kit prior to 10X Chromium sequencing; a minimum of 50 ng DNA was submitted for 10X sequencing. HMW DNA was sheared into an average fragment size between 12-20 kb in a Megaruptor 3 system with speed setting 30. Sheared DNA was purified by solid-phase reversible immobilisation using AMPure PB beads with a 1.8X ratio of beads to sample to remove the shorter fragments and concentrate the DNA sample. The concentration of the sheared and purified DNA was assessed using a Nanodrop spectrophotometer and Qubit Fluorometer and Qubit dsDNA High Sensitivity Assay kit. Fragment size distribution was evaluated by running the sample on the FemtoPulse system.

Pacific Biosciences HiFi circular consensus and 10X Genomics read cloud sequencing libraries were constructed according to the manufacturers’ instructions. Sequencing was performed by the Scientific Operations core at the WSI on Pacific Biosciences SEQUEL II and Illumina HiSeq X instruments. Hi-C data were generated using the Arima v2.0 kit and sequenced on HiSeq X.

Assembly was carried out with Hifiasm ( Cheng *et al*., 2021), haplotypic duplication was identified and removed with purge_dups ( Guan *et al*., 2020). The assembly was polished with the 10X Genomics Illumina data by aligning to the assembly with longranger align, calling variants with freebayes ( Garrison & Marth, 2012). One round of the Illumina polishing was applied. Scaffolding with Hi-C data ( Rao *et al*., 2014) was carried out with SALSA2 ( Ghurye *et al*., 2019). The assembly was checked for contamination and corrected using the gEVAL system ( Chow *et al*., 2016) as described previously ( Howe *et al*., 2021). Manual curation was performed using gEVAL, HiGlass ( Kerpedjiev *et al*., 2018) and Pretext. The mitochondrial genome was assembled using MitoHiFi ( Uliano-Silva *et al*., 2021). The genome was analysed and BUSCO scores generated within the BlobToolKit environment ( Challis *et al*., 2020). Table 3 contains a list of all software tool versions used, where appropriate.

**Table 3.** Software tools used.

Software tool	Version	Source
Hifiasm	0.12	Cheng *et al*., 2021
purge_dups	1.2.3	Guan *et al*., 2020
longranger	2.2.2	https://support.10xgenomics.com/genome-exome/software/pipelines/latest/advanced/ other-pipelines
freebayes	v1.3.1-17- gaa2ace8	Garrison & Marth, 2012
MitoHiFi	2.0	Uliano-Silva *et al*., 2021
SALSA2	2.2	Ghurye *et al*., 2019
gEVAL	N/A	Chow *et al*., 2016
HiGlass	1.11.6	Kerpedjiev *et al*., 2018
PretextView	0.2.x	https://github.com/wtsi-hpag/PretextView
BlobToolKit	2.6.2	Challis *et al*., 2020

The materials that have contributed to this genome note have been supplied by a Darwin Tree of Life Partner. The submission of materials by a Darwin Tree of Life Partner is subject to the Darwin Tree of Life Project Sampling Code of Practice. By agreeing with and signing up to the Sampling Code of Practice, the Darwin Tree of Life Partner agrees they will meet the legal and ethical requirements and standards set out within this document in respect of all samples acquired for, and supplied to, the Darwin Tree of Life Project. Each transfer of samples is further undertaken according to a Research Collaboration Agreement or Material Transfer Agreement entered into by the Darwin Tree of Life Partner, Genome Research Limited (operating as the Wellcome Sanger Institute), and in some circumstances other Darwin Tree of Life collaborators.

## Data availability

European Nucleotide Archive: Lineus longissimus (bootlace worm). Accession number PRJEB45185: https://identifiers.org/ena.embl:PRJEB45185

The genome sequence is released openly for reuse. The *L. longissimus* genome sequencing initiative is part of the Darwin Tree of Life (DToL) project. All raw sequence data and the assembly have been deposited in INSDC databases. The genome will be annotated and presented through the Ensembl pipeline at the European Bioinformatics Institute. Raw data and assembly accession identifiers are reported in Table 1.
