# Supplementary material for: The genome sequence of the bootlace worm, Lineus longissimus (Gunnerus, 1770)
Source: Wellcome Open Res. 2021 Oct 14;6:272. [Version 1] doi: 10.12688/wellcomeopenres.17193.1 (PMC8593623; doi:10.12688/wellcomeopenres.17193.1)
Supplement: Peer review history

# Peer review: Reviewer response for version 1

**DOI:** 10.21956/wellcomeopenres.18996.r46958

**Published:** 2021-11-15

**Authors:** Ferdinand Marletaz

**Affiliations:** 1 Centre for Life's Origins and Evolution, Department of Genetics, Evolution and Environment, University College London, London, UK

I think this provides an accurate report of the genome assembly of the nemertean *Lineus longissimus*. 

 "Their extensibility results in part from their unsegmented body form, where the coleom is limited to a rhynchocoel associated with the eversible proboscis "

 -> I think it is not so opportune to link extensibility with reduced coelome, a lot of animals with reduced or absent coelom and they are not so extensibly (planarians, etc...), so I would suggest to rephrase

 "Phylum Nemertea is placed as sister to either Mollusca or Annelida within Eutrochozoa in the Spiralia (Struck & Fisse, 2008). "

 -> I think you should cite some more recent references and discuss the alternative phylogenetic positions as suggested by Dr. Martin-Duran. 

 It would be interesting to mention the estimated polymorphism and the estimated genome size. 

 I would also recommend to cite Luo et al. (2018) ^1^ in the introduction as it is the first nermetean genome characterised.

Are sufficient details of methods and materials provided to allow replication by others?

Yes

Is the rationale for creating the dataset(s) clearly described?

Yes

Are the datasets clearly presented in a useable and accessible format?

Yes

Are the protocols appropriate and is the work technically sound?

Yes

Reviewer Expertise:

animal phylogeny, regulatory and comparative genomics, spiralians, evodevo

I confirm that I have read this submission and believe that I have an appropriate level of expertise to confirm that it is of an acceptable scientific standard.

## References

[1] : Nemertean and phoronid genomes reveal lophotrochozoan evolution and the origin of bilaterian heads. *Nat Ecol Evol* .2018;2(1) : 10.1038/s41559-017-0389-y 141-151 10.1038/s41559-017-0389-y 29203924

# Peer review: Reviewer response for version 1

**DOI:** 10.21956/wellcomeopenres.18996.r46445

**Published:** 2021-10-19

**Authors:** Jose Maria Martin-Duran

**Affiliations:** 1 School of Biological and Chemical Sciences, Queen Mary University of London, London, UK

This is a clear and concise report on the sequencing of Lineus longissiums genome, an iconic species in the European biota. Methods and the resulting assembly are gold-standard. I only have three minor comments:

- L. longissimus might actually be the longest animal species ever recorded (see https://www.guinnessworldrecords.com/world-records/longest-animal)
- The phylogenetic position of Nemerteans is contentious. Recent phylogenomic analyses place them either as sister to Platyhelminthes (https://doi.org/10.1016/j.cub.2018.11.042 ^1^) or Lophophorata (https://doi.org/10.1016/j.cub.2015.06.068 ^2^), in addition to the previous phylogenetic associations with Annelida and Mollusca.
- Importantly, this is not the first Nemertean genome to be sequenced (https://www.nature.com/articles/s41559-017-0389-y ^3^). In any case, it is not unusual that nemertean sequences do not appear in public databases.

Are sufficient details of methods and materials provided to allow replication by others?

Yes

Is the rationale for creating the dataset(s) clearly described?

Yes

Are the datasets clearly presented in a useable and accessible format?

Yes

Are the protocols appropriate and is the work technically sound?

Yes

Reviewer Expertise:

evolutionary developmental biology, evolutionary genomics

I confirm that I have read this submission and believe that I have an appropriate level of expertise to confirm that it is of an acceptable scientific standard.

## References

[1] : A New Spiralian Phylogeny Places the Enigmatic Arrow Worms among Gnathiferans. *Current Biology* .2019;29(2) : 10.1016/j.cub.2018.11.042 312-318.e3 10.1016/j.cub.2018.11.042 30639106

[2] : Spiralian phylogeny informs the evolution of microscopic lineages. *Curr Biol* .2015;25(15) : 10.1016/j.cub.2015.06.068 2000-6 10.1016/j.cub.2015.06.068 26212884

[3] : Nemertean and phoronid genomes reveal lophotrochozoan evolution and the origin of bilaterian heads. *Nat Ecol Evol* .2018;2(1) : 10.1038/s41559-017-0389-y 141-151 10.1038/s41559-017-0389-y 29203924
